# The role of material and psychosocial resources in explaining socioeconomic inequalities in diet: A structural equation modelling approach

**DOI:** 10.1016/j.ssmph.2022.101025

**Published:** 2022-01-16

**Authors:** Jody C. Hoenink, Wilma Waterlander, Joline W.J. Beulens, Joreintje D. Mackenbach

**Affiliations:** a Amsterdam UMC, Vrije Universiteit Amsterdam, Department of Epidemiology and Data Science, Amsterdam Public Health Research Institute, De Boelelaan, 1117, Amsterdam, the Netherlands; b Upstream Team, www.upstreamteam.nl, Amsterdam UMC, the Netherlands; c Amsterdam UMC, University of Amsterdam, Department of Public and Occupational Health, Amsterdam Public Health Research Institute, Meibergdreef 9, Amsterdam, the Netherlands; d Julius Center for Health Sciences and Primary Care, University Medical Center Utrecht, Utrecht University, Utrecht, the Netherlands

**Keywords:** Explanatory factors, Adults, Diet, Socioeconomic differences, SES, Inequity

## Abstract

We examined whether material and psychosocial resources may explain socioeconomic differences in diet quality. Cross-sectional survey data from 1461 Dutch adults (42.5 (SD 13.7) years on average and 64% female) on socio-demographics, diet quality, psychosocial factors and perceptions of and objective healthiness of the food environment were used in a structural equation model to examine mediating pathways. Indicators for socioeconomic position (SEP) were income, educational, and occupational level and the 2015 Dutch Healthy Diet (DHD15) index assessed diet quality. Material resources included food expenditure, perceptions of healthy food accessibility and healthfulness of the food retail environment. Psychosocial resources were cooking skills, resilience to unhealthy food environments, insensitivity to food cues and healthy eating habits. Higher SEP was associated with better diet quality; B_education_ 8.5 (95%CI 6.7; 10.3), B_income_ 5.8 (95%CI 3.7; 7.8) and B_occupation_ 7.5 (95%CI 5.5; 9.4). Material resources did not mediate the association between SEP and diet quality and neither did the psychosocial resources insensitivity to food cues and eating habits. Cooking skills mediated between 13.3% and 19.0% and resilience to unhealthy food environments mediated between 5.9% and 8.6% of the relation between SEP and the DHD15-index. Individual-level factors such as cooking skills can only explain a small proportion of the SEP differences in diet quality. On top of other psychosocial and material resources not included in this study, it is likely that structural factors outside the individual, such as financial, work and living circumstances also play an important role.

## Highlights

• Higher income, educational level and occupation associated with better dietary quality.
• Material resources did not explain socioeconomic inequalities in diet.
• Some, but not all, psychosocial resources explained socioeconomic inequalities in diet.
• 20% of the association between socioeconomic position and diet was explained.
• Structural factors outside the individual likely play an important role.

## 1 Introduction

The prevalence of non-communicable diseases (NCDs), adverse outcomes from NCDs, and risk factors for NCDs are unevenly distributed across socioeconomic gradients (Sommer et al., 2015). For example, evidence shows that individuals with a lower socioeconomic position (SEP) are at an increased risk of having cardiovascular disease (Sommer et al., 2015), more often have obesity (Wang & Beydoun, 2007), and tend to have lower quality diets (Darmon & Drewnowski, 2008) compared to individuals with a higher SEP. The Black report marked a milestone in understanding how social conditions shape health inequalities (Black, 1982). Several theories have been posited to explain socioeconomic inequalities in health. The life course perspective recognizes that both health and SEP in later life are not independent of health experiences, exposures, and economic resources and inequalities from earlier in the life course (e.g. during early childhood) (Corna, 2013). Another theory, the social causation perspective, posits that socioeconomic conditions affect health largely through diverse material, psychosocial and behavioural risk factors (Schmitz & Pförtner, 2018).

Indeed, studies found that material (e.g. receiving public benefits and having financial problems), psychosocial (e.g. social capital and anxiety) and behavioural (e.g. smoking habits and alcohol consumption) factors can explain socioeconomic inequalities in self-rated health (Schmitz & Pförtner, 2018; Van Lenthe et al., 2004) and mortality (Skalická et al., 2009). Evidence also suggests that material and psychosocial factors work through behavioural factors to influence health (Schmitz & Pförtner, 2018; Van Lenthe et al., 2004). As such, it can be hypothesized that material and psychosocial factors (partly) explain socioeconomic inequalities in dietary behaviours. Material resources that may facilitate adherence to a healthier diet include sufficient food budgets, access to healthful food stores and owning cooking equipment. Psychosocial resources derived from a higher SEP may comprise food preparation skills, social support, and resilience to unhealthy temptations in the food environment.

The explanatory power of material and psychosocial resources may depend on the SEP indicator under study and the extent to which material and psychosocial factors interact. While education, income and occupation all represent the general concept of SEP, they also provide specific resources unique to the indicator, which may translate into differential associations with dietary outcomes (Galobardes et al., 2001). As such, the explanatory power of material resources such as food budget may be stronger when income is used as an indicator of SEP. However, taking into account only one explanatory factor could potentially overestimate single pathways, which does not improve our understanding of their contribution in relation to other factors (Moor et al., 2017). As evidence suggests that material resources may be able to compensate the lack of psychosocial resources (Dendup et al., 2021; Mackenbach et al., 2015), it is especially important to explore the relative contribution of material and psychosocial resources in dietary inequalities.

To the best of our knowledge, no previous studies have examined the simultaneous mediating role of psychosocial and material resources in socioeconomic inequalities in diet. Some studies have shown that psychosocial resources such as better cooking skills, resilience to unhealthy food environments, sensitivity to food cues and healthy eating habits (i.e. habitual eating behaviours that one has developed over time (Stok et al., 2018)) are related to dietary behaviours or obesity (Anglé et al., 2009; Hartmann et al., 2013; Lowe et al., 2009; McGowan et al., 2016). There is also evidence that some of these psychosocial factors are differentially distributed among SEP groups (Adams et al., 2015; Spinosa et al., 2019). A number of studies explored the role of (subjective and objective) food cost or affordability as material explanations for socioeconomic inequalities in dietary behaviours (Aggarwal et al., 2011; Beydoun & Wang, 2008; Dijkstra et al., 2015; Hoenink et al., 2020; Pechey & Monsivais, 2016), but few studies focused on access to healthy food retail as a material resource (Ball et al., 2006; Inglis et al., 2008).

In this study we aimed to examine the individual and combined mediating role of material and psychosocial resources in the association between SEP and diet quality, separately for three SEP indicators. While we hypothesize that all SEP indicators are connected to material and psychosocial resources, we expect that income is most strongly related to material resources and educational level and occupation more strongly to psychosocial resources (Fliesser et al., 2017).

## 2 Methods

### 2.1 Study population

This study used survey data from the cross-sectional ‘Eet & Leef’ study on eating and lifestyle behaviours among adults from the general population (18–65 years) living in urban areas in the Netherlands. Participants were recruited through a stepwise recruitment approach. First, postal invitations were sent to ∼21,500 randomly selected home addresses in the twenty largest cities of the Netherlands. Also, Facebook and Instagram campaigns were used. In addition, several lower educated men who participated in previous studies received an invitation to participate in the current study via e-mail. Inclusion criteria were: understanding the Dutch language, having access to a computer with internet and having an email-address.

In total, 2533 participants registered for the study of whom 2434 were eligible to participate and invited to complete three parts of a survey. Overall, 1492 participants completed all three parts of the survey. Questions covered different domains regarding the determinants of food choices, of which the current study used data on socio-demographic and socioeconomic characteristics, diet quality, psychosocial resources and perceptions and use of the food environment. Participants who completed all three questionnaires received a gift voucher of €7,50. The study design and procedures were approved by the Medical Ethics Review Committee of VU University Medical Centre (no. 2019.307) and all participants gave written informed consent.

### 2.2 Outcome

Dietary intake data as assessed by the Dutch Healthy Diet Food Frequency Questionnaire (DHD-FFQ) was used to calculate adherence to the Dutch Health Diet index 2015 (DHD15-index) (van Lee, Geelen, et al., 2016). The DHD-FFQ is a short screener questionnaire which, in a previous study, was validated against a 180-item FFQ combined with a 24 h urinary sodium excretion value (van Lee, Feskens, et al., 2016). This validation showed that the DHD15-index derived from the DHD-FFQ was acceptable in ranking individuals but relatively poor in the absolute individual assessment of diet quality. Energy intake was also estimated. The DHD15-index was calculated as described by Looman et al. (Looman et al., 2017), resulting in a total score ranging from 0 to 150 points, with higher scores indicating better adherence to the guidelines. Participants were excluded if they had implausible energy intake levels (Banna et al., 2017) (n = 31). This resulted in an analytical sample of 1461 participants.

### 2.3 Determinants

Participants' answered questions about their educational level, occupation and net household income. *Education* was assessed using the question ‘What is your highest educational attainment?’ and consisted of seven options varying from having not completed any formal education to university degree. Due to the distribution of the data and to facilitate comparison between the three indicators, educational level was categorized into low/medium educational level and high educational level. Low/medium educational level included those who completed no education, primary education, secondary education or intermediate vocational education. High educational level included those who completed higher professional education (College/University).

*Occupation* was assessed using the question ‘What is your profession/has been your profession?‘. The open-ended answers to this question were classified into four categories according to the International Standard Classification of Occupations 2008 (ISCO-08) (Ganzeboom, 2010). Because less than 50 participants could be categorized in the first category, occupation was dichotomized by combining the first two skill levels (low/medium skill-level occupation) and the last two skill levels (high skill-level occupation). Low/medium skill-level occupations include those involving the performance of simple and routine physical or manual tasks or tasks such as operating machinery. High skill-level occupations include those involving the performance of complex technical and practical skills or those that require complex problem-solving, decision-making and creativity (Ganzeboom, 2010).

*Net household income* was assessed with the question ‘What is your net household income (after tax deduction) per month?’ and consisted of 5 answering options ranging from ‘€0–1200’ to ‘more than €4000’ per month. *Household equivalent income* was calculated by multiplying overall household income with weighting factors according to household members using the OECD-modified scale (Organisation for Economic Co-operation and Development, 2020). The monthly household equivalent income was dichotomized into low/medium (≤€1733) and high (>€1733) income based on the median individual income in the Netherlands (Central Bureau of Statistics, 2019).

### 2.4 Mediators

The survey included several questionnaires assessing constructs that could potentially mediate the association between SEP and diet quality. A selection of constructs was made based on: 1) whether these factors were resources supporting healthy eating and 2) the psychometric properties of the questionnaires. Only questionnaires with acceptable psychometric properties within this study sample were selected. The structural validity was measured using either exploratory factor analysis (EFA) or confirmatory factor analysis (CFA), and the internal consistency was assessed using the Cronbach's alpha (≥0.7 was regarded as acceptable).

Material resources included the average weekly food budget, perceptions of healthy food accessibility and the objectively-measured healthy food retail environment around the home. Psychosocial resources were those relating to individual differences and social relationships that potentially have beneficial effects on dietary intake, including cooking skills, resilience to unhealthy food environments in general, insensitivity to food cues and several eating habits.

#### 2.4.1 Material resources

*Perceived access to healthy food* was assessed using the Perceived Food Environment questionnaire (Carbonneau et al., 2017). This questionnaire consisted of six questions relating to the accessibility of healthy foods and three questions relating to the limited accessibility of unhealthy foods measured on a 5-point Likert scale. CFA in the current study sample did not confirm the proposed factor structure of nine items loading onto two factors. The internal consistency of the three items related to access to unhealthy foods was unacceptable. Based on the results of an EFA, two items on the accessibility of healthy foods were removed. The internal consistency of the remaining four items relating to the accessibility of healthy foods were acceptable (Cronbach's alpha of 0.84). The included items can be found in Supplementary Table 1.

*Objective access to a healthy food retail environment* was assessed through objective data on the presence of healthy and unhealthy food retailers within a 10 min walk from the participants’ home address according to an 800-m Euclidean buffer. Using validated commercial food environment data from Locatus (Canalia et al., 2020), the percentage of healthier food retailers of the total amount of food retailers was calculated using the modified Retail Food Environment Index (mRFEI). The classification by Timmermans et al. was used to classify food-retailers as healthy or unhealthy (Timmermans et al., 2018). The food retail environment of participants with no food retailers around their home (n = 38 in this analytical sample) was considered as healthy (i.e. value 1).

*Food budget* was assessed by asking participants what their average weekly expenses on groceries were (6 answering options, ranging from 0 to 25€ to >200€). The weekly *household equivalent food budget* was calculated by using the upper end of the six answering options (250€ for the last answering option) and weighting these according to the number of household members using the OECD-modified scale (Organisation for Economic Co-operation and Development, 2020).

#### 2.4.2 Psychosocial resources

*Cooking skills* were assessed using six questions (on a 5-point Likert scale) on the subscale ‘food preparation skills’ from the Food Literacy Questionnaire (Poelman et al., 2018). A higher score indicated that participants had better cooking skills. CFA did not confirm the six items loading onto one factor. Based on EFA results, one item was removed (see Supplementary Table 1 for the included items)The internal consistency was acceptable (Cronbach's alpha 0.77).

*Resilience to unhealthy food environments* was measured using a single item question on a 5-point Likert scale; ‘Do you eat healthy, even when the food environment makes this difficult?‘. A higher score indicated more resilience to unhealthy food environments.

*Sensitivity to food cues* was assessed using the abbreviated, Dutch translation of an 11-item Power of Food Scale questionnaire (De Vet et al., 2014) based on a selection approved by the authors of the original scale (Cappelleri et al., 2009). CFA analysis in the full sample did not confirm that the 11 items loaded onto one factor. Based on the EFA results, two items were removed and the remaining items loaded onto one factor. The internal consistency of the 9-item Power of Food Scale was acceptable (Cronbach's alpha 0.88). The items in the questionnaire were reversed in order for a higher score to indicate less sensitivity to food cues (protective effect).

*Eating behaviours* were assessed using an adapted, Dutch translation of the 18 item Three-Factor Eating Questionnaire (Anglé et al., 2009). This questionnaire assessed cognitive restraint, uncontrolled eating and emotional eating on a 4-point Likert scale. Restrained eaters exert cognitive effort to control their food intake, uncontrolled eaters tend to overeat with the feeling of being out of control and emotional eaters tend to eat in response to negative emotions (Anglé et al., 2009; Bryant et al., 2019). CFA in the full study sample could not confirm the proposed factor structure of 18 items loading onto three factors. Based on the EFA results, four items were removed and the remaining items loaded onto three factors (Supplementary Table 1). The internal consistency of the items belonging to the factors uncontrolled eating (Cronbach's alpha 0.86; 6 items), emotional eating (Cronbach's alpha 0.93; 3 items) and cognitive restraint (Cronbach's alpha 0.82; 5 items) were acceptable. The factors uncontrolled and emotional eating were recoded and renamed to ‘controlled eating’ and ‘indifferent eating’ in order to reflect a resource towards healthy eating.

### 2.5 Covariates

Information regarding participants’ age, sex, partner (yes/no), number of children in the household, height and weight, and energy intake were assessed through questionnaires. Body Mass Index (BMI) was calculated as self-reported weight in kilograms divided by the square height in meters.

### 2.6 Statistical analyses

Descriptive statistics were computed for all variables using frequencies and percentages for categorical variables, means with standard deviations for normally distributed continuous variables, and median with interquartile range (IQR) for skewed continuous variables. Item-nonresponse was found for the variables education (1%), income (8%), occupation (7%) and mRFEI (3%).

**Fig. 1:**
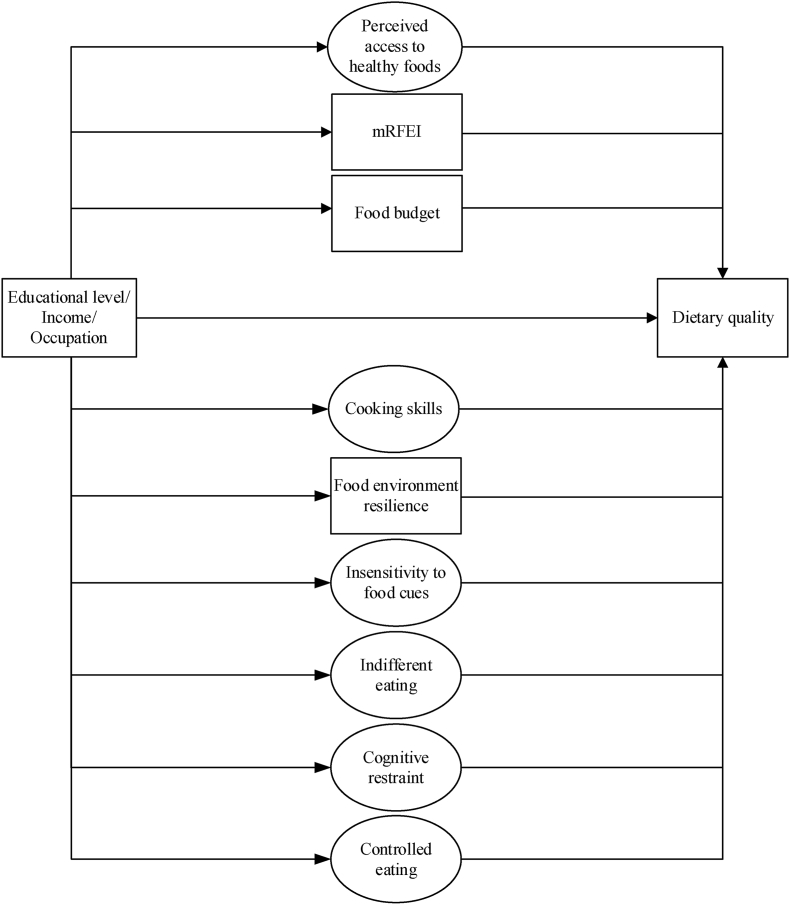
Final parallel mediation models in SEM. Circles represent latent variables and rectangles represent measured variables. Dotted rectangles/circles represent material resources and dashed squares/circles represent psychosocial resources.

Structural Equation Modelling (SEM) in STATA v14.1 was used to investigate the mediating role of food-related material and psychosocial resources in the association between SEP indicators and dietary intake. Complete case analyses were conducted and the Maximum Likelihood (ML) approach was used. The construction of SEM models is generally an iterative process which consists of determining indicators to latent variables (the measurement model) and the expression and quality of the relations between (latent) variables (the structural model). Supplementary Fig. 1 displays the proposed mediation model. EFA did not support the measurement models for material resources combined and psychosocial resources combined as latent variables explained by the sumscores of the different questionnaires. As such, all material and psychosocial resources were treated as individual (latent) mediating variables in the structural models (Fig. 1). We first investigated single mediation models, with the association between a SEP indicator and dietary intake (c-path; total effect), the association between a SEP indicator and a mediator (a-path), the association between a mediator and dietary intake (b-path) and the association between a SEP indicator and dietary intake adjusted for a mediator (c’-path; indirect effect). Then, parallel mediation analysis including all material and psychosocial resources was performed. The proportion mediated was calculated (indirect effect/total effect), but only if *1)* significant mediation was found, *2)* the total (c path) and indirect (a-path * b-path) effect had the same direction and *3)* if the indirect effect was smaller than the total effect. For all mediation models, a bootstrapped 95% confidence interval (1000 bootstrap resamples and seed number 1234) around the indirect effect was calculated. Age, sex, partner, children in the household, BMI and energy intake were included as covariates. Significance was set when the 95% confidence intervals did not include zero. We report the model fit for the single and parallel mediation models. Goodness of fit of the models was defined based on the comparative fit index (CFI), root mean square error of approximation (RMSEA), standardized root mean square residual (SRMSR) and a χ2 test. An RMSEA value of <0.05 indicates good fit, <0.08 indicates acceptable fit and 0.08–0.10 indicates neither a good nor a bad fit. A good fit for CFI relates to a value greater than 0.95, while a value greater than 0.90 indicates a satisfactory fit. A good fit for SRMR is a value smaller than 0.05, and a value between 0.05 and 0.10 is an acceptable fit (Schreiber et al., 2006).

## 3 Results

### 3.1 Descriptives

The mean age of participants was 42.5 (SD 13.7) years and the majority of participants were female (64.1%) (Table 1). In total, 42.6%, 31.5% and 34.3% of participants were considered to have a low/medium level of education, household equivalent income or occupation, respectively. Participants with a high SEP had higher mean DHD15-index scores compared to participants with a low/medium SEP.

**Table 1:** Characteristics of the study population by educational level, occupation and household equivalent income.

	Low/medium education N = 617	High education N = 832	Low/medium income N = 424	High income N = 924	Low/medium skilled occupation N = 468	High skilled occupation N = 897	Total N = 1461
Socio-demographics							
Age; mean years (SD)	44.2 (14.7)	41.3 (12.7)	41.3 (15.1)	43.0 (12.7)	43.2 (14.3)	42.9 (12.9)	42.5 (13.7)
Sex; % female	62.9	64.9	67.5	61.5	63.9	63.9	64.1
BMI; mean (SD)	25.9 (5.1)	24.0 (4.1)	25.3 (5.5)	24.6 (4.2)	26.0 (5.1)	24.3 (4.3)	24.8 (4.6)
Partner; % yes	61.4	71.4	55.7	71.5	62.4	71.1	66.9
Children in household; % no children	73.6	74.9	74.5	74.5	73.1	73.8	74.5
Energy intake; mean (SD)	1526.1 (560.9)	1517.0 (472.3)	1567.1 (587.2)	1518.0 (479.8)	1531.1 (558.6)	1517.2 (476.7)	1521.1 (512.7)
**Material resources**							
Perceived access to healthy foods (range 1–5); median p25-p75	4.0; 3.8–4.5	4.0; 3.8–4.6	4.0; 3.5–4.5	4.0; 3.8–4.5	4.0; 3.8–4.5	4.0; 3.8–4.8	4.0; 3.8–4.5
mRFEI (range 0–100); mean % (SD)	34.9 (19.0)	32.8 (20.2)	33.1 (17.2)	33.7 (20.5)	35.4 (19.3)	32.9 (20.1)	33.7 (19.7)
Household equivalent food budget (range 10–250); mean (SD)	74.6 (33.1)	85.1 (35.6)	62.4 (29.2)	90.2 (34.0)	73.7 (33.1)	85.7 (34.8)	80.5 (35.0)
**Psychosocial resources**							
Cooking skills (range 1–5); median p25-p75	3.8; 3.4–4.4	4.2; 3.6–4.6	4.0; 3.4–4.4	4.2; 3.6–4.6	3.8; 3.4–4.4	4.2; 3.6–4.6	4.0; 3.4–4.6
Resilience to unhealthy food environment (range 1–5); mean (SD)	3.3 (0.9)	3.5 (0.9)	3.2 (1.0)	3.4 (0.8)	3.2 (1.0)	3.5 (0.8)	3.4 (0.9)
Insensitivity to food cues (range 1–5); mean (SD)	2.8 (0.7)	2.9 (0.7)	2.8 (0.7)	2.9 (0.7)	2.8 (0.7)	2.9 (0.7)	2.8 (0.7)
Indifferent eating (range 1–4); mean (SD)	2.9 (0.8)	2.9 (0.8)	2.8 (0.9)	2.9 (0.8)	2.9 (0.8)	2.9 (0.8)	2.9 (0.8)
Controlled eating (range 1–4); mean (SD)	2.9 (0.6)	3.0 (0.5)	2.9 (0.6)	3.0 (0.5)	2.9 (0.6)	3.0 (0.5)	2.9 (0.6)
Cognitive restraint (range 1–4); mean (SD)	2.3 (0.6)	2.3 (0.6)	2.2 (0.6)	2.4 (0.6)	2.3 (0.6)	2.3 (0.6)	2.3 (0.6)
**Dietary intake**							
DHD15-index (range 0–150); mean (SD)	91.1 (18.5)	100.2 (17.3)	91.3 (18.6)	98.3 (17.8)	91.0 (18.4)	99.4 (17.6)	96.3 (18.3)

Abbreviations: BMI; Body Mass Index (kg/m^2^), SD; Standard Deviation, IQR; Interquartile Range mRFEI; modified Retail Food Environment Index.

Regarding the material resources, the median perceived access to healthy foods was 4.0 (IQR 0.8) and 30 percent of the retail food environment around participants’ home were considered healthy (Table 1). These resources were approximately equally distributed among low and high SEP participants. The mean household equivalent food budget for the overall population was €80.5 (SD 35.0) per week, with higher food budgets for high SEP participants. Regarding psychosocial resources, participants had a median score of 4.0 (IQR 1.2) on the cooking skills questionnaire and a mean score of between 2.3 and 3.4 on the questionnaires related to resilience to unhealthy food environments resilience, insensitivity to food cues and eating habits, with hardly any differences between low/medium and high SEP participants.

### 3.2 Model fit

Supplementary Figure 2 displays the measurement models for the latent material and psychosocial factors. With regards to the structural model, theparallel mediation models (model in Fig. 1) including all nine resources for the three SEP indicators was acceptable for the model fit indices RMSEA and SRMR (RMSEA = 0.06 and SRMR = 0.10), but not the CFI (CFI = 0.86) or χ2 test. To improve the goodness of fit, parallel mediation models only including significant mediators were performed as a sensitivity analysis. While all measures improved, the p-value of the χ2 remained significant which may be due to the large sample size. All goodness of fit statistics for the single and parallel mediation models are presented in Supplementary Table 2.

### 3.3 SEP inequalities in diet quality

Participants with a high education scored 8.5 (95%CI 6.7; 10.3) points higher on the DHD15-index compared to participants with a low/medium education. For the SEP proxies income and occupation this difference was 5.8 (95%CI 3.7; 7.8) and 7.5 (95%CI 5.5; 9.3), respectively (Table 2; total effect).

**Table 2:** Results of the parallel mediation models regarding the role of material and psychosocial resources in the association between the three SEP indicators and the DHD15-index.

Independent variables	Mediators	Dependent variable	Total effect (c-path)		Direct effect (c’-path)		Indirect effect (a-path x b-path)		Proportion mediated
			β	95%CI	β	95%CI	β	Bootstrap 95%CI	$\frac{\mathrm{AB}}{\left(C^{\prime }+\mathrm{AB}\right)}$
Educational level	Cooking skills	DHD15-index	**8.5**	**6.7; 10.3**	**6.9**	**5.1; 8.7**	**1.1**	**0.6; 1.7**	14.1%
	Environment resilience						**0.5**	**0.2; 0.9**	5.9%
	Insensitivity to food cues						0.0	−0.1; 0.2	N/A
	Indifferent eating						−0.2	−0.4; 0.1	N/A
	Controlled eating						0.0	−0.2; 0.3	N/A
	Cognitive restraint						−0.0	−0.1; 0.1	N/A
	Food budget						0.0	−0.3; 0.3	N/A
	mRFEI						−0.1	−0.2; 0.1	N/A
	Access to healthy foods						−0.0	−0.1; 0.1	N/A
Income	Cooking skills		**5.8**	**3.7; 7.8**	**4.3**	**2.2; 6.4**	**1.1**	**0.5; 1.7**	19.0%
	Environment resilience						**0.5**	**0.1; 0.9**	8.6%
	Insensitivity to food cues						0.1	−0.1; 0.3	N/A
	Indifferent eating						−0.1	−0.3; 0.1	N/A
	Controlled eating						0.1	−0.2; 0.4	N/A
	Cognitive restraint						−0.1	−0.2; 0.1	N/A
	Food budget						−0.0	−0.8; 0.7	N/A
	mRFEI						0.0	−0.1; 0.1	N/A
	Access to healthy foods						−0.1	−0.2; 0.1	N/A
Occupation	Cooking skills		**7.5**	**5.5; 9.4**	**6.0**	**4.1; 7.9**	**1.0**	**0.4; 1.6**	13.3%
	Environment resilience						**0.5**	**0.1; 0.8**	6.7%
	Insensitivity to food cues						0.1	−0.1; 0.2	N/A
	Indifferent eating						−0.2	−0.5; 0.1	N/A
	Controlled eating						0.1	−0.2; 0.4	N/A
	Cognitive restraint						0.0	−0.1; 0.1	N/A
	Food budget						0.0	−0.3; 0.4	N/A
	mRFEI						−0.1	−0.2; 0.1	N/A
	Access to healthy foods						0.0	−0.1; 0.1	N/A

Abbreviations: **β**; unstandardized regression coefficient, mRFEI; modified Retail Food Environment Index.

### 3.4 Mediation by material and psychosocial resources

No mediation by material resources was found in the relation between SEP and the DHD15-index (Supplementary Table 3). Similarly, the psychosocial resources insensitivity to food cues and the three eating habit factors did not mediate the association between SEP and the DHD15-index, while cooking skills and food environment resilience did mediate this association. The same results are found in the parallel mediation models in Table 2.

The indirect effect of SEP on the DHD15-index via cooking skills corrected for all other material and psychosocial resources varied between 1.0 (95%CI_occupation_ 0.4; 1.6) and 1.1 (e.g. 95%CI_education_ 0.6; 1.7) (Table 2). The indirect effect of SEP on the DHD15-index via food environment resilience corrected for all other material and psychosocial resources was 0.5 (e.g. 95%CI_occupation_ 0.1; 0.8). The proportion mediated for cooking skills varied between 13.3% and 19.0% and the proportion mediated for food environment resilience varied between 5.9% and 8.6% (Table 2; proportion mediated). The strongest mediation effects were found for the SEP indicator household equivalent income. The total indirect effect of SEP on the DHD15-index via all nine resources varied between 1.5 (95%CI_income_ 0.3; 2.6) and 1.6 (95%CI_education_ 0.7; 2.4 and 95%CI_occupation_ 0.6; 2.4), resulting in a proportion mediated of between 18.8% and 25.9%. These results are similar for the parallel mediation models only including significant mediators with acceptable model fit indices (Supplementary Table 4).

## 4 Discussion

We investigated the mediating role of material and psychosocial resources in the association between SEP and diet quality. None of the studied material resources and only two psychosocial resources mediated the association between SEP and diet quality. Together, cooking skills and food environment resilience accounted for approximately 20% of the association between SEP and diet quality, which highlights the need to look for more systemic factors that could explain socio-economic inequalities in diet.

As shown in previous studies (Darmon & Drewnowski, 2015; Giskes et al., 2010), individuals with a higher SEP had better quality diets. Only the psychosocial resources cooking skills and resilience to the unhealthy food environment partly explained SEP inequalities in diet quality. We believe this is because skills and knowledge-based resources are more strongly related to SEP than cognition-based resources such as food cue reactivity (Ball et al., 2006; McKinnon et al., 2014; McLeod et al., 2011; Sugisawa et al., 2015). Furthermore, the present study findings suggest that the three socioeconomic indicators included in this study have similar associations with diet quality through material and psychosocial resources. It is possible that socioeconomic indicators on a different level – e.g. childhood SEP or neighbourhood SEP – show more disparate effects on dietary behaviours (Lallukka et al., 2007). Another possibility is that individual-level SEP indicators work similarly in less diverse study populations compared to more diverse populations as sociodemographic variables such as ethnicity and sex can influence SEP (Krieger et al., 1997). Thus, the predominantly White population included in the current study may explain the similar results across SEP indicators.

Whereas there is consistent evidence that the cost of food (Aggarwal et al., 2011; Beydoun & Wang, 2008; Dijkstra et al., 2015; Hoenink et al., 2020; Pechey & Monsivais, 2016) and some evidence that the objectively measured accessibility to healthy foods (Ball et al., 2006) partly explain dietary inequalities, we found no evidence for a mediating role of these material resources in the association between SEP and diet quality. This may be due to the Dutch context; the Netherlands is highly urbanized and has relatively good geographic access to food (Karampour et al., 2016). In addition, foods are relatively affordable compared to other European countries (Eurostat, 2020). As such, food-related material resources may be accountable for less of the socioeconomic dietary disparities in the Netherlands than in other contexts. Indeed, in a previous study we showed that the cost of food only explained approximately 5% of the association between SEP and diet quality in the Netherlands (Hoenink et al., 2020). This is much lower than studies conducted in the United Kingdom and the United States where the proportion mediated ranged from 31% to 76% (Aggarwal et al., 2011; Pechey & Monsivais, 2016).

While food prices, nutrition knowledge, cooking skills and unhealthy food environment resilience may help explain socioeconomic inequalities in dietary behaviours, most of the association between SEP and diet quality still remains unexplained. This could be attributed to the fact that the mediating factors under study are individual-level and diet-specific factors. It is likely that broader factors, other than those directly relating to dietary behaviour, play an important role. The unequal distribution of income, food, education and power may influence diet through attentional, emotional and material consequences. For example, housing insecurity can lead to emotional responses such as stress and poor sleep, which in turn may lead to poorer dietary choices through attentional consequences (Laraia et al., 2017). As such, the factors previous studies found to explain socioeconomic inequalities in health (Schmitz & Pförtner, 2018; Skalická et al., 2009), may also partly explain socioeconomic inequalities in diet (e.g. financial problems, receiving public benefits, type of health insurance, housing tenure, control beliefs, social participation, anxiety and self-esteem).

Thus, the SEP/behaviour relationship is complex and most likely requires considerations of broader system factors such as the community, environment and public policy. Langellier et al. illustrate the utility of complex systems methods for unravelling wider underlying mechanisms that shape population dietary patterns as well understanding decision support for diet and nutrition policy and model validation (Langellier et al., 2019). Here it is important to recognize that relations between factors are generally not linear and static, but are in fact dynamic and respond to feedback. For example, Hammond et al., suggest how social influence interacts with other mechanisms such as social capital and social stress generated by social relations, to influence diet (Hammond, 2010). Furthermore, such complex system methods may be better able to take into account the life course perspective of how relationships to larger social, economic, and historical contexts may influence both the continuity and change of dietary behaviours.

The notion that broader-level factors influence specific behaviours which may lead to socioeconomic inequalities should also be taken into account in the design of preventative interventions. While interventions aimed at individual factors (e.g. providing cooking lessons) tend to have some effect (Kroeze et al., 2006), they may actually increase socioeconomic inequalities due to their dependence on individual “agency”. Furthermore, these types of interventions are often not sustainable or scalable. In contrast, it takes no individual agency to benefit from population-level approaches such as a sugar sweetened beverage tax (Adams et al., 2016). These population-level interventions may also have a more lasting effect on behaviour change compared to individual-level interventions because they can become incorporated into structures, systems, policies, and sociocultural norms (Larson & Story, 2009). However, it may be necessary to address the root causes of social inequalities in order to close the gap between the diet quality of those with the highest versus the lowest socioeconomic positions. For example, given the large market power, corporate wealth and income distribution of the global soft drink market, a recent study suggests to explore potential government levers such as market concentration, market power and shareholder primacy (Wood et al., 2021). Kumanyika's framework for increasing equity impact in obesity prevention does not only include recommendations on diet-related factors such as reducing the promotion of unhealthy products or increasing nutrition assistance programs, but also broad-level factors such as empowering communities, and reducing threats to personal safety and discrimination (Kumanyika, 2019).

Strengths of the study include the relatively large sample recruited from different areas throughout the Netherlands. Another strength is the incorporation of multiple potential mediators and assessing their exploratory role simultaneously. However, a limitation of the study is that only a selective set of variables were available to represent material and psychosocial resources. Another limitation is that the present findings are based on cross-sectional data, limiting interpretations about the directions of the mediating pathways. Furthermore, we used self-reported food frequency questionnaire data, which can lead to under- or overreported dietary intake. The last limitation is that individuals with a lower SEP had a lower response to the study even though the cumulative response rate of 59% was similar to those in other mail surveys (Inglis et al., 2008; Sugisawa et al., 2015). Caution is needed when generalizing the results to those with the lowest SEP. Potential strategies that could be employed to include more individuals with a lower SEP may include tailoring questionnaires to the target group, using existing networks to reach the target group (e.g. food banks and community centres) and providing incentives (Stuber et al., 2020).

### 4.1 Conclusion

In conclusion, individual-level factors such as cooking skills and resilience to the unhealthy food environment can only explain a small proportion of the SEP inequalities in diet quality. Material resources and the psychosocial resources insensitivity to food cues and eating habits do not seem to explain SEP inequalities in diet quality in the Dutch context. However, the explanatory mechanisms of social inequalities in diet may have to be sought in the wider financial, work and living circumstances that differ between socioeconomic groups.

## Financial support

The ‘Eet & Leef’ study, and the work of JDM, is funded by an 10.13039/501100003246*NWO VENI* *grant* on “Making the healthy choice easier – role of the local food environment” (grant number 451-17-032). JCH and JDM are further funded by the 10.13039/501100002996Netherlands Heart Foundation (Hartstichting) and the 10.13039/501100001826Netherlands Organisation for Health Research and Development (ZonMw) through the Supreme Nudge (CVON2016–04) project.

## Authorship

JDM set up the ‘Eet & Leef’ study and collected the data. JCH, JWJB, WW and JDM designed the current study. JCH conducted the formal analyses and drafter the paper. JCH, JWJB, WW and JDM interpreted the results and all authors reviewed and edited the manuscript.

## Ethical standard disclosure

The ‘Eet & Leef’ study was performed in line with the principles of the Declaration of Helsinki. The study was approved by the Medical Ethics Review Committee of the VU University Medical Centre (no. 2019.307).

## Declaration of competing interest

The authors have declared that no competing interests exist.
