# Supplementary material for: The role of material and psychosocial resources in explaining socioeconomic inequalities in diet: A structural equation modelling approach
Source: SSM Popul Health. 2022 Jan 16;17:101025. doi: 10.1016/j.ssmph.2022.101025 (PMC8783096; doi:10.1016/j.ssmph.2022.101025)
Supplement: Multimedia component 1 [file mmc1.docx]

# Supplementary file

Supplementary Table 1. Questions and items belonging to the various questionnaires

| **Item** | **Question** |
| --- | --- |
| **Perceived access to healthy foods** | |
| 1 | I consider that the quantity of healthy foods offered by my main food retailer is sufficient |
| 2 | I consider that the variety of healthy foods offered by my main food retailer is sufficient |
| 3 | I consider that the quality of healthy foods offered by my main food retailer is acceptable |
| 4 | I consider that the cost of healthy foods offered by my main food retailer is affordable |
| **Cooking skills** | |
| 1 | Are you able to prepare fresh vegetables in different ways?  For example cooking, steaming or stir frying, or in different dishes? |
| 2 | Do you find it difficult to prepare a meal with more than five fresh ingredients? |
| 4 | Are you able to prepare fresh fish in different ways?  For example grilling, pan frying or stewing, or in different dishes? |
| 5 | Are you able to prepare a meal using fresh ingredients?  So without pre-packed and processed foods? |
| 6 | Are you able to see, smell or feel the quality of fresh foods?  For example of meat, fish or fruit? |
| **Insensitivity to food cues** | |
| 1 | I find myself thinking about food even when I’m not physically hungry |
| 2 | I get more pleasure from eating than I do from almost anything else |
| 3 | If I see or smell a food I like, I get a powerful urge to have some |
| 4 | When I know a delicious food is available, I can’t help myself from thinking about having some |
| 5 | When I eat delicious food I focus a lot on how good it tastes |
| 6 | Hearing someone describe a great meal makes me really want to have something to eat |
| 8 | I think I enjoy eating a lot more than most other people |
| 9 | It seems like I have food on my mind a lot |
| 11 | Before I eat a favorite food my mouth tends to flood with saliva |
| **Eating behaviours – indifferent eating** | |
| 11 | When I feel blue, I often overeat |
| 12 | When I feel anxious, I find myself eating |
| 13 | When I feel lonely, I console myself by eating |
| **Eating behaviours – cognitive restraint** | |
| 1 | I deliberately take small helpings as means of controlling my weight |
| 2 | I consciously hold back at meals in order not to gain weight |
| 3 | I do not eat some foods because they make me fat |
| 17 | How likely are you to consciously eat less than you want? |
| 18 | How often do you deliberately hold back at meals in order not to gain weight? |
| **Eating behaviours – controlled eating** | |
| 5 | I get so hungry that my stomach often seems like a bottomless pit |
| 6 | I am always hungry so it is hard for me to stop eating before I finish the food  on my plate |
| 7 | I am always hungry enough to eat at any time |
| 8 | When I see a real delicacy, I often get so hungry that I have to eat right away |
| 9 | Being with someone who is eating often makes me hungry enough to eat  also |
| 15 | How often do you feel hungry? |

Supplementary Figure 1. Hypothesized measurement model connecting SEP indicators with dietary quality. Circles represent latent variables and rectangles represent measured variables.

Supplementary Figure 2. Measurement model for the included material and psychosocial resources. Circles represent latent variables and rectangles represent measured variables.

Supplementary Table 2. Model fit indices for the single and parallel mediation models

| Independent variable | Mediator | Outcome | χ2 | p-value χ2 | RMSEA | CFI | SRMR |
| --- | --- | --- | --- | --- | --- | --- | --- |
| Education | Perceived access to healthy foods | DHD15-index | 115.97 | 0.00 | 0.05 | 0.97 | 0.02 |
|  | Cooking skills |  | 149.52 | 0.00 | 0.05 | 0.95 | 0.02 |
|  | Insensitivity to food cues |  | 729.48 | 0.00 | 0.07 | 0.90 | 0.04 |
|  | Indifferent eating |  | 28.51 | 0.01 | 0.02 | 1.00 | 0.01 |
|  | Controlled eating |  | 449.12 | 0.00 | 0.08 | 0.91 | 0.03 |
|  | Cognitive restraint |  | 334.98 | 0.00 | 0.08 | 0.91 | 0.04 |
|  | Cooking skills and food environment resilience |  | 278.6 | 0.00 | 0.06 | 0.91 | 0.05 |
|  | All potential mediators |  | 4149.8 | 0.00 | 0.06 | 0.86 | 0.10 |
| Income | Perceived access to healthy foods |  | 98.95 | 0.00 | 0.05 | 0.98 | 0.02 |
|  | Cooking skills |  | 120.33 | 0.00 | 0.04 | 0.96 | 0.02 |
|  | Insensitivity to food cues |  | 718.29 | 0.00 | 0.07 | 0.89 | 0.04 |
|  | Indifferent eating |  | 31.21 | 0.01 | 0.03 | 1.00 | 0.01 |
|  | Controlled eating |  | 427.68 | 0.00 | 0.08 | 0.90 | 0.03 |
|  | Cognitive restraint |  | 330.19 | 0.00 | 0.08 | 0.91 | 0.04 |
|  | Cooking skills and food environment resilience |  | 243.1 | 0.00 | 0.06 | 0.92 | 0.04 |
|  | All potential mediators |  | 3951.6 | 0.00 | 0.06 | 0.86 | 0.10 |
| Occupation | Perceived access to healthy foods |  | 120.42 | 0.00 | 0.05 | 0.97 | 0.02 |
|  | Cooking skills |  | 118.86 | 0.00 | 0.04 | 0.96 | 0.02 |
|  | Insensitivity to food cues |  | 694.78 | 0.00 | 0.07 | 0.89 | 0.04 |
|  | Indifferent eating |  | 29.48 | 0.01 | 0.03 | 1.0 | 0.01 |
|  | Controlled eating |  | 412.51 | 0.00 | 0.07 | 0.91 | 0.03 |
|  | Cognitive restraint |  | 350.5 | 0.00 | 0.08 | 0.90 | 0.04 |
|  | Cooking skills and food environment resilience |  | 240.2 | 0.00 | 0.06 | 0.92 | 0.04 |
|  | All potential mediators |  | 3941.0 | 0.00 | 0.06 | 0.86 | 0.10 |

The model fit indices for the single mediation models including measured factors (i.e., mRFEI, food budget and food environment resilience) are not displayed because these models had zero degrees of freedom and therefore represented a saturated model with perfect goodness of fit (i.e. RMSEA = 0.00, CFI = 1.00 and SRMR = 0.00)

Supplementary Table 3. Results of the single mediation models regarding the role of material and psychosocial resources in the association between the three SEP indicators and the DHD15-index

| **Independent variables** | **Mediators** | **Dependent variable** | **SEP on resources**  **(a-path)** | | **Resources on diet quality**  **(b-path)** | | **Total effect**  **(c-path)** | | **Direct effect**  **(c’-path)** | | **Indirect effect**  **(c-path –**  **c’-path)** | | **Proportion mediated** | |
| --- | --- | --- | --- | --- | --- | --- | --- | --- | --- | --- | --- | --- | --- | --- |
|  |  |  | **β** | **95%CI** | **β** | **95%CI** | **β** | **95%CI** | **β** | **95%CI** | **β** | **Bootstrap 95%CI** | | $\frac{\mathrm{AB}}{(C^{'}+AB)}$ |
| Educational level | Access to healthy foods | DHD15-index | 0.0 | -0.0; 0.1 | 0.0 | -0.0; 0.0 | **8.6** | **6.8; 10.4** | **8.5** | **6.7; 10.3** | 0.0 | -0.0; 0.0 | | N/A |
|  | mRFEI |  | **-0.1** | **-0.1; -0.0** | 0.0 | -0.0; 0.1 |  |  | **8.6** | **6.8; 10.4** | -0.0 | -0.1; 0.1 | | N/A |
|  | Food budget |  | **0.2** | **0.1; 0.2** | 0.0 | -0.0; 0.1 |  |  | **8.4** | **6.6; 10.2** | 0.2 | -0.2; 0.5 | | N/A |
|  | Cooking skills |  | **0.2** | **0.1; 0.2** | **0.2** | **0.2; 0.3** |  |  | **7.1** | **5.3; 8.9** | **1.4** | **0.7; 2.1** | | 16.3% |
|  | Food environment resilience |  | **0.1** | **0.0; 0.1** | **0.2** | **0.2; 0.3** |  |  | **7.8** | **6.0; 9.6** | **0.7** | **0.2; 1.2** | | 8.2% |
|  | Insensitivity to food cues |  | -0.0 | -0.1; 0.0 | 0.0 | -0.0; 0.1 |  |  | **8.6** | **6.8; 10.4** | 0.0 | -0.0; 0.0 | | N/A |
|  | Indifferent eating |  | 0.0 | -0.1; 0.1 | -0.0 | -0.1; 0.0 |  |  | **8.6** | **6.8; 10.4** | -0.0 | -0.0; 0.0 | | N/A |
|  | Controlled eating |  | **0.1** | **0.0; 0.1** | -0.1 | -0.1; 0.0 |  |  | **8.7** | **6.9; 10.5** | -0.1 | -0.3; 0.0 | | N/A |
|  | Cognitive restraint |  | 0.0 | -0.1; 0.1 | **0.1** | **0.1; 0.2** |  |  | **8.5** | **6.8; 10.3** | 0.0 | -0.3; 0.3 | | N/A |
| Income | Access to healthy foods |  | **0.1** | **0.0; 0.1** | 0.0 | -0.0; 0.0 | **5.8** | **3.8; 7.8** | **5.7** | **3.7; 7.8** | 0.0 | -0.1; 0.2 | | N/A |
|  | mRFEI |  | 0.0 | -0.0; 0.1 | 0.0 | -0.0; 0.1 |  |  | **5.7** | **3.6; 7.7** | 0.0 | -0.0; 0.0 | | N/A |
|  | Food budget |  | **0.4** | **0.3; 0.4** | 0.0 | -0.0; 0.1 |  |  | **5.5** | **3.4; 7.7** | 0.2 | -0.6; 0.9 | | N/A |
|  | Cooking skills |  | **0.1** | **0.1; 0.2** | **0.2** | **0.2; 0.3** |  |  | **4.7** | **2.7; 6.6** | **1.1** | **0.4; 1.8** | | 19.0% |
|  | Food environment resilience |  | **0.1** | **0.0; 0.1** | **0.2** | **0.2; 0.3** |  |  | **5.0** | **3.1; 7.0** | **0.8** | **0.3; 1.3** | | 13.8% |
|  | Insensitivity to food cues |  | -0.0 | -0.1; 0.0 | 0.0 | -0.0; 0.1 |  |  | **5.8** | **3.8; 7.8** | -0.1 | -0.2; 0.1 | | N/A |
|  | Indifferent eating |  | 0.0 | -0.0; 0.1 | -0.0 | -0.1; 0.0 |  |  | **5.8** | **3.8; 7.8** | -0.0 | -0.0; 0.0 | | N/A |
|  | Controlled eating |  | 0.0 | -0.0; 0.1 | -0.1 | -0.1; 0.0 |  |  | **5.8** | **3.8; 7.8** | -0.1 | -0.2; 0.1 | | N/A |
|  | Cognitive restraint |  | 0.0 | -0.0; 0.1 | **0.1** | **0.1; 0.2** |  |  | **5.7** | **3.7; 7.7** | 0.1 | -0.2; 0.4 | | N/A |
| Occupation | Access to healthy foods |  | -0.0 | -0.1; 0.0 | 0.0 | -0.0; 0.1 | **7.4** | **5.5; 9.3** | **7.4** | **5.5; 9.5** | -0.0 | -0.1; 0.0 | | N/A |
|  | mRFEI |  | **-0.1** | **-0.1; -0.0** | 0.0 | -0.0; 0.1 |  |  | **7.5** | **5.6; 9.4** | -0.0 | -0.2; 0.1 | | N/A |
|  | Food budget |  | **0.2** | **0.1; 0.2** | 0.0 | -0.0; 0.1 |  |  | **7.3** | **5.4; 9.3** | 0.2 | -0.1; 0.5 | | N/A |
|  | Cooking skills |  | **0.1** | **0.1;0.2** | **0.2** | **0.2; 0.3** |  |  | **6.2** | **4.3; 8.1** | **1.2** | **0.6; 1.8** | | 16.2% |
|  | Food environment resilience |  | **0.1** | **0.0; 0.1** | **0.2** | **0.2; 0.3** |  |  | **6.7** | **4.8; 8.6** | **0.7** | **0.3; 1.2** | | 9.5% |
|  | Insensitivity to food cues |  | -0.0 | -0.1; 0.0 | 0.0 | -0.0; 0.1 |  |  | **7.5** | **5.5; 9.4** | -0.1 | -0.2; 0.1 | | N/A |
|  | Indifferent eating |  | -0.0 | -0.1; 0.0 | -0.0 | -0.1; 0.0 |  |  | **7.4** | **5.5; 9.3** | 0.0 | -0.1; 0.1 | | N/A |
|  | Controlled eating |  | **0.1** | **0.0; 0.1** | **-0.1** | **-0.1; -0.0** |  |  | **7.6** | **5.7; 9.5** | -0.2 | -0.4; 0.0 | | N/A |
|  | Cognitive restraint |  | 0.0 | -0.0; 0.1 | **0.1** | **0.1; 0.2** |  |  | **7.3** | **5.4; 9.2** | 0.1 | -0.2; 0.4 | | N/A |

Models were adjusted for age, sex, partner, BMI, number of children in the household and energy intake

Abbreviations: B; standardised beta regression coefficient, CI; confidence interval, N/A; Not Applicable

Bold values indicate significance as the 95% confidence interval does not include zero

Supplementary Table 4. The combined mediating role of psychosocial mediators in the association between the three SEP indicators and the DHD15-index

| **Independent variables** | **Mediators** | **Dependent variable** | **SEP on psychosocial resources**  **(a-path)** | | **Psychosocial resources on diet quality**  **(b-path)** | | **Total effect**  **(c-path)** | | **Direct effect**  **(c’-path)** | | **Indirect effect**  **(c-path – c’-path)** | | **Proportion mediated** |
| --- | --- | --- | --- | --- | --- | --- | --- | --- | --- | --- | --- | --- | --- |
|  |  |  | **β** | **95%CI** | **β** | **95%CI** | **β** | **95%CI** | **β** | **95%CI** | **β** | **Bootstrap 95%CI** | $\frac{\mathrm{AB}}{(C^{'}+AB)}$ |
| Educational level | Cooking skills | DHD15-index | **0.2** | **0.1; 0.2** | **0.2** | **0.1; 0.2** | **8.6** | **6.8; 10.3** | **6.9** | **5.1; 8.7** | **1.1** | **0.5; 1.6** | 12.8% |
|  | Food environment resilience |  | **0.1** | **0.0; 0.1** | **0.2** | **0.1; 0.2** |  |  |  |  | **0.6** | **0.2; 1.0** | 7.0% |
| Income | Cooking skills |  | **0.1** | **0.1; 0.2** | **0.2** | **0.1; 0.2** | **5.8** | **3.8; 7.8** | **4.3** | **2.4; 6.3** | **0.9** | **0.3; 1.4** | 15.5% |
|  | Food environment resilience |  | **0.1** | **0.0; 0.1** | **0.2** | **0.1; 0.2** |  |  |  |  | **0.6** | **0.2; 1.0** | 10.3% |
| Occupation | Cooking skills |  | **0.1** | **0.1; 0.2** | **0.2** | **0.1; 0.2** | **7.4** | **5.5; 9.3** | **5.9** | **4.0; 7.8** | **0.9** | **0.4; 1.5** | 12.2% |
|  | Food environment resilience |  | **0.1** | **0.0; 0.1** | **0.2** | **0.1; 0.2** |  |  |  |  | **0.6** | **0.2; 1.0** | 8.1% |

Models were adjusted for age, sex, partner, BMI, number of children in the household and energy intake

Abbreviations: B; standardised beta regression coefficient, CI; confidence interval, N/A; Not Applicable

Bold values indicate significance as the 95% confidence interval does not include zero
